# Impact of the interval between neoadjuvant immunochemotherapy and surgery on surgical–pathological outcomes in non-small cell lung cancer

**DOI:** 10.3389/fonc.2022.909726

**Published:** 2022-09-07

**Authors:** Jiawei Chen, Hongsheng Deng, Jiaxi He, Zhufeng Wang, Shuben Li

**Affiliations:** ^1^ Department of Thoracic Surgery and Oncology, State Key Laboratory of Respiratory Disease, National Clinical Research Center for Respiratory Disease, Guangzhou Institute of Respiratory Health, The First Affiliated Hospital of Guangzhou Medical University, Guangzhou, China; ^2^ National Clinical Research Center for Respiratory Disease, State Key Laboratory of Respiratory Disease, Guangzhou Institute of Respiratory Health, The First Affiliated Hospital of Guangzhou Medical University, Guangzhou, China

**Keywords:** time-to-surgery (TTS) interval, surgery, surgical safety, pathological outcomes, neoadjuvant immunotherapy

## Abstract

**Introduction:**

The interval between neoadjuvant immunochemotherapy and surgery in patients with non-small cell lung cancer (NSCLC) has not been well characterized. This study investigated the association between the time-to-surgery (TTS) interval and surgical–pathological outcomes.

**Method:**

Clinical data of patients who received neoadjuvant immun-ochemotherapy followed by surgery for NSCLC between January 2019 and September 2021 were collected. The patients were divided into three groups based on TTS interval: the early-surgery group (ESG), the standard-surgery group (SSG), and the delayed-surgery group (DSG). The primary outcomes were objective response rate (ORR), major pathological response (MPR), and pathological complete response (pCR). The secondary endpoint was surgical outcome.

**Results:**

Of the 171 patients, 16 (9.4%) received surgery in ≤28 days, 49 (28.7%) received surgery within 29–42 days, and 106 (61.9%) received surgery in ≥43 days after neoadjuvant immunochemotherapy, with a median TTS of 46 days. The postoperative drainage of the ESG group (455.1 ml) was significantly less than that of the SSG group (680.7 ml) and the DSG group (846.5 ml; p = 0.037). However, the TTS interval did not influence the duration of the operation (*P* = 0.54), the extent of intraoperative bleeding (*P* = 0.60), or the length of postoperative hospital stay (*P* = 0.17). The ORR was observed in 69%, 51%, and 56% of patients in the ESG, the SSG, and the DSG, respectively (*P* = 0.46), and MPR occurred in 50%, 47%, and 58% (*P* = 0.38) of patients in the ESG, the SSG, and the DSG, respectively. Similarly, no statistically significant difference was found for pCR (ESG: 31%; SSG: 27%; DSG: 42%; *P* = 0.14).

**Conclusion:**

This retrospective study indicated that TTS exerts no significant effect on the feasibility and safety of surgery in the neoadjuvant immunochemotherapy setting of NSCLC. Analysis of the TTS interval revealed a tendency for delayed surgery to be associated with a pathological response in NSCLC, although this association was not statistically significant.

## Introduction

Lung cancer remains the leading cause of cancer-related death worldwide (1, 2), accounting for 24% and 23% of cancer-related deaths in men and women, respectively. In the past few years, preoperative programmed cell death protein 1 (PD-1) or its ligand, PD-L1, alone or combined chemotherapy, has been investigated in several clinical trials of non-small cell lung cancer (NSCLC) (3–7). This treatment pattern, which can effectively reduce the size of locally advanced tumors and improve their pathological response (8), is recommended for early-stage NSCLC and resectable, locally advanced NSCLC.

Previously, Liu et al. (9) established a spontaneously metastatic cancer model in mice with 4T1.2 and E0771 cancer cell types and demonstrated that a short duration (4–5 days) between the first administration of neoadjuvant immunotherapy and resection of the primary tumor was necessary for optimal efficacy and that extending this duration (≥10 days) or giving neoadjuvant immunotherapy too close to surgery (≤2 days) reduced immunotherapy efficacy. These results suggest that the time-to-surgery (TTS) interval should be carefully considered to achieve a better oncological outcome. However, limited data exist to determine the optimal TTS in NSCLC, particularly in the neoadjuvant immunochemotherapy setting. According to the latest expert consensus (10), it is recommended that surgery be performed 4–6 weeks after the last cycle of neoadjuvant immunochemotherapy. Nevertheless, no research has validated this recommendation or thoroughly investigated the association between the TTS interval and pathological downstaging. Furthermore, whether a long TTS interval increases surgical difficulty has not been established.

We thus conducted a population-based, real-world, retrospective study to evaluate whether TTS impacts surgical and pathological outcomes.

## Method

Patients who had biopsy-confirmed, clinical stage II/III NSCLC and who received neoadjuvant immunochemotherapy followed by surgery for NSCLC between January 2019 and September 2021 were identified from the clinical data. The preoperative and postoperative staging were evaluated in accordance with the eighth American Joint Committee on Cancer (AJCC) and lung cancer staging manuals on the tumor, node, and metastasis (TNM) staging systems (11). The TTS interval was defined as the time from the day of the last treatment cycle to the day of surgery. The patients were divided into three groups based on TTS: the early-surgery group (ESG: TTS ≤28 days; n = 16), the standard-surgery group (SSG: TTS 29–42 days; n = 49), and the delayed-surgery group (DSG: TTS ≥43 days; n = 106). The primary outcomes were the objective response rate (ORR), the major pathological response (MPR) rate, and the pathological complete response (pCR) rate. MPR was defined as 10% or fewer viable tumor cells in the resected primary tumor, and the pCR was defined as the removal of carinal tissues and dissected lymph nodes without any viable tumor. The surgical outcomes included operation time, intraoperative bleeding, postoperative drainage, and hospital stay. Multivariable regression analysis was conducted to adjust for confounders such as tumor size, histology, and surgical procedures. Odds ratios for pathological and surgical outcomes were estimated by multivariate regression using robust standard errors. Results are reported as odds ratios with a 95% CI. All statistical analyses were conducted using the SPSS v. 23 software (IBM Corp., Armonk, NY, USA).

## Results

### Baseline characteristics

A total of 171 patients were enrolled in the study, all of whom underwent routine staging, including chest computed tomography (CT) and endoscopy for histological biopsy. Most of the patients (n = 126, 73.68%) had stage IIIA or IIIB disease. The average tumor diameter prior to immunochemotherapy was 5.34 cm (a range of 1.6–15.2 cm). Detailed baseline characteristics and surgical and oncological outcomes are summarized in **Table 1**. Twenty patients had delayed administration of neoadjuvant immunotherapy, mostly due to the COVID-19 pandemic and their physical condition. Because of the COVID-19 pandemic, there were 15 patients who had delayed treatment, with five patients having delayed immunochemotherapy due to their physical condition. Moreover, no patient experienced a dose reduction. The TTS intervals after the last cycle of immunochemotherapy ranged from 15 to 107 days, with a median TTS of 46 days. There were 16 (9.4%) patients with TTS ≤28 days, 49 (28.7%) patients with TTS between 29 and 42 days, and 106 (61.9%) patients with TTS ≥43 days.

**Table 1:** Baseline characteristics of patients with NSCLC.

Covariate	Full Sample (n = 171)	Time ≤28 d (n = 16)	Time 2,942 d (n =4 9)	Time ≥43 d (n = 106)	*P* value
**Age**					0.43
Mean (SD)	60.8 (8.7)	58.3 (9)	61.6 (7.6)	60.8 (9.2)	
Median (Min, Max)	62 (25, 84)	57.5 (40, 73)	62 (45, 84)	62 (25, 77)	
**Sex**					0.91
Female	26 (15)	3 (19)	7 (14)	16 (15)	
Male	145 (85)	13 (81)	42 (86)	90 (85)	
**BMI**					0.37
Mean (SD)	23 (2.6)	22.1 (1.7)	23.2 (3.4)	23.1 (2.3)	
Median (Min, Max)	23 (16.7, 32.8)	21.3 (19.3, 25.2)	22.7 (16.7, 32.8)	23.1 (18.2, 29.2)	
**Cycles of treatment**					0.19
≤2	73 (43)	3 (19)	26 (53)	44 (42)	
3	42 (25)	7 (44)	8 (16)	27 (25)	
4	32 (19)	5 (31)	9 (18)	18 (17)	
5	16 (9)	1 (6)	5 (10)	10 (9)	
≥6	8 (5)	0 (0)	1 (2)	7 (7)	
**Surgery safety**					0.78
Open	20 (12)	2 (12)	7 (14)	11 (10)	
Others	151 (88)	14 (88)	42 (86)	95 (90)	
**Surgery type**					0.29
Lobectomy	145 (85)	12 (75)	40 (82)	93 (88)	
Sleeve lobectomy	19 (11)	2 (12)	6 (12)	11 (10)	
Others	7 (4)	2 (12)	3 (6)	2 (2)	
**Stage**					0.33
Before 3	26 (15)	2 (12)	9 (18)	15 (14)	
3a	65 (38)	4 (25)	20 (41)	41 (39)	
3b	61 (36)	9 (56)	12 (24)	40 (38)	
>3b	19 (11)	1 (6)	8 (16)	10 (9)	

### Surgical outcomes and their relationship between TTS

Of the 171 patients, 145 (85%) received lobectomy, 19 (11%) underwent sleeve lobectomy, and seven were (4%) treated with other types of lung resection. The vast majority of the patients (n = 151, 88.3%) received minimally invasive surgery, and 20 patients were converted to thoracotomy, mostly due to serious pleural adhesions and pulmonary arterial hemorrhage. In the overall cohort, the mean surgical time was 189.3 min (a range of 90–475 min). The mean intraoperative bleeding volume was 172.3 ml (a range of 5–4,000 ml). The average postoperative drainage was 660.8 ml (a range of 10–3,830 ml). The average postoperative hospital stay was 5.4days (a range of 2–21 days). No 90-day surgical-related mortality was recorded. In this analysis, postoperative drainage was associated with the TTS interval. The drainage volume of the ESG group was significantly lower than that of the SSG group and the DSG group (ESG, 455.1 vs. SSG, 680.7 vs. DSG, 846.5; P = 0.037). However, the TTS showed no influence on the duration of operation (*P* = 0.54), intraoperative bleeding volume (*P*=0.6), or postoperative hospital (*P*=0.17).

### Pathological response and the relationship with TTS

The percentages of patients who achieved pCR in the SSG, ESG, and DSG were 27%, 31%, and 42%, respectively (*P* = 0.14), with a similar pattern occurring with MPR (DSG, 58% vs. ESG, 50% vs. SSG, 47%; *P* = 0.38). Although not statistically significant, a slightly higher proportion of patients achieved pCR and MPR in the DSG. ORR showed no difference across the three groups (DSG, 56% vs. ESG, 69% vs. SSG, 51%; *P* = 0.46; **Table 2**). Moreover, multivariable regression analysis was performed to adjust for confounders, including tumor size and histology, and surgical procedures (**Table 3**).

**Table 2:** Pathological and surgical outcomes of patients.

Covariate	Time ≤28 d (n = 16)	Time 29–42 d (n = 49)	Time ≥43 d (n = 106)	p-value
**Surgery time (min)**				0.54
Mean (sd)	202.2 (88.1)	189.2 (91.3)	176.5 (60.2)	
Median (Min, Max)	175 (105, 475)	162.5 (85, 520)	160 (90, 350)	
**Blood loss (ml)**				0.6
Mean (sd)	199.3 (437.4)	152.9 (275)	164.7 (470.9)	
Median (Min, Max)	40 (20, 1,500)	50 (10, 1,100)	30 (5, 4,000)	
**Postoperative drainage (ml)**				**0.037**
Mean (sd)	455.1 (345.2)	680.7 (505.8)	846.5 (693)	
Median (Min, Max)	400 (22, 1,120)	505 (10, 2,000)	660 (10, 3,830)	
**Postoperative hospital stay (days)**				0.17
Mean (sd)	4.8 (1.7)	5.4 (2.5)	6 (2.9)	
Median (Min, Max)	4 (3, 8)	5 (2, 14)	5 (3, 21)	
**MPR**				0.38
MPR	8 (50)	23 (47)	62 (58)	
Others	8 (50)	26 (53)	44 (42)	
**PCR**				0.14
pCR	5 (31)	13 (27)	45 (42)	
Others	11 (69)	36 (73)	61 (58)	
**ORR**				0.46
ORR	11 (69)	25 (51)	59 (56)	
Others	5 (31)	24 (49)	47 (44)	

The bold values meaned the postoperative drainage showed the satistical significance with TTS.

**Table 3:** Multivariable regression analysis was performed to adjust for confounders including tumor size and histology, and surgical procedures.

Outcomes	TTS	OR (95% CI)	*P*-value
**ORR**	≤28	–	–
	29–42	0.31 (0.05–1.29)	0.13
	≥43	0.37 (0.07–1.47)	0.19
**MPR**	≤28	–	–
	29–42	0.91 (0.26–3.19)	0.88
	≥43	1.26 (0.39–4.08)	0.69
**pCR**	≤28	–	–
	29–42	0.45 (0.11–1.85)	0.26
	≥43	0.96 (0.28–3.55)	0.95
**Postoperative hospital stay**	≤28	–	–
	29–42	1.11 (0.46–2.71)	0.81
	≥43	1.60 (0.70–3.65)	0.26
**Operative time**	≤28	–	–
	29–42	7.33 × 10^−11^ (3.33 × 10^−21^–1.61 × 10)	0.06
	≥43	2.25 × 10^−4^ (4.87 × 10^–14^–1.04 × 10^6^)	0.46
**Intraoperative bleeding**	≤28	–	–
	29–42	1.38 × 10^17^ (1.27 × 10^–13^–1.50 × 10^47^)	0.26
	≥43	3.36 × 10^9^ (1.60 × 10^-19^–7.09×10^37^)	0.51
**Postoperat ive drainage**	≤28	–	–
	29–42	1.43 × 10^70^ (1.67 × 10^-36^–1.23×10^176^)	0.193
	≥43	1.94 × 10^74^ (1.11 × 10^−25^—3.39 × 10^173^)	0.141

## Discussion

The current study indicated that early lung resection within 28 days is safe, as the postoperative morbidity, mortality, safety of surgery, and pathological outcomes were similar across the different study groups.

There is a paucity of data available on optimal TTS after neoadjuvant immunochemotherapy in NSCLC, with more evidence relating to other cancers. Bausys et al. (12) reported that an interval of 30 days or less between the completion of neoadjuvant treatment and surgery significantly correlated with a higher MPR. Omarini et al. (13) and Sanford et al. (14) concluded that a short interval between neoadjuvant chemotherapy (NAC) and surgery might be more effective for breast cancer patients. In contrast, Du et al. (15) demonstrated that a prolonged interval (>8 weeks) contributed to a higher pathological outcome in rectal cancer. Moreover, Terzi (16) reported that extending the interval between neoadjuvant chemoradiation (NCRT) and surgery from 8 to 12 weeks led to a 2-fold increase in the pCR rate. Similarly, a series of studies (17–19) examining the NCRT pattern in esophageal cancer consistently found a prolonged interval between NCRT and esophagectomy to be significantly associated with a higher rate of pCR. The same trend was observed in this study. Although not statistically significant, a tendency toward higher MPR and pCR was also found in patients undergoing delayed surgery.

According to Liu et al. (9), adequate time is necessary for the antitumor response to develop after the administration of immune checkpoint inhibitors. Additionally, patients need time to recover from the short-term side effects of therapy. In the trial by Amaria et al. (20), most of the enrolled patients required a 9-week interval between the three doses of neoadjuvant immunotherapy and surgery due to toxicity issues. In terms of surgical difficulty and safety in the setting of neoadjuvant immunotherapy, Liang et al. (21) found that it is more difficult to perform lung resection after neoadjuvant immunotherapy due to serious tissue edema and increased capillary fragility, which may increase the risk of bleeding and blood loss. Ma et al. (22) indicated that neoadjuvant therapy may increase the chance of structural damage, and in their study, the intraoperative bleeding of cases that received neoadjuvant chemotherapy plus osophagectomy was higher than that for those that received osophagectomy alone. To date, the relationship between surgical outcomes and TTS remains unclear. This study showed that, although not statistically significant, a tendency for increased intraoperative bleeding occurred in patients undergoing delayed surgery, likely because a longer TTS usually correlates with structural damage. The COVID-19 pandemic has been responsible for the widespread delay of surgeries. According to several previous studies (23–25), COVID-19 was significantly associated with postoperative complications and a higher rate of mortality. Lei et al. (25) reported that the mortality rate of patients with COVID-19 in their study was 20.5%, and 44.5% of patients required intensive care in an intensive care unit after surgery. Another study (26) indicated that surgery should be delayed after COVID-19 to potentially prevent postoperative complications. Furthermore, during the waves of the current COVID-19 pandemic, the TTS has increased due to deferred surgical resection as a result of operating room closures. Therefore, a considerable number of patients receiving neoadjuvant therapy may experience delayed operations. Although TTS does not affect surgical indicators in the overall population, a few cases suggest that prolonged TTS may still affect the outcome of the operation, as shown in the following patients. First, in this study, there were seven patients with intraoperative bleeding of more than 1,000 ml after neoadjuvant immunochemotherapy. All were treated with surgery in ≥43 days, with mean TTS intervals of 48 days (a range of 43–72 days) after the last cycle of neoadjuvant immunochemotherapy. Of the seven patients with NSCLC, three had stage IIIA, two had stage IIIB, and two had stage IIB. Moreover, five patients received lobectomy and two underwent sleeve lobectomy. Serious pleural adhesion occurred in all of the patients. Thus, randomized controlled trials are required to verify these findings.

This study has several limitations. First, the decisions made for TTS were possibly dependent on personal experience and tumor radiological response. Second, this study was retrospective with a limited sample size. Considering this is a retrospective study, selection bias should be considered.

## Conclusion

In this study, the TTS interval showed no significant effect on surgical feasibility or safety in the neoadjuvant immunochemotherapy setting of NSCLC. Pathological outcomes, although not statistically significant, showed a trend in which delayed surgery contributed to a better pathological response. Further studies with larger sample sizes are needed for validation of these findings.

## Data availability statement

The original contributions presented in the study are included in the article/supplementary material. Further inquiries can be directed to the corresponding author.

## Ethics Statement

Written informed consent was obtained from the individual(s) for the publication of any potentially identifiable images or data included in this article.

## Author contributions

JC: conception and design of the study and drafting the article. HD: analysis of data, visualization of data, contributing cases who provided administration support. ZW: analysis of data and interpretation of data. JH: revised the article critically. SL: conception and design of the study, revised the article critically, administration support, and general supervision of the research group. All authors listed have made a substantial, direct, and intellectual contribution to the work and approved it for publication.

## Conflict of interest

The authors declare that the research was conducted in the absence of any commercial or financial relationships that could be construed as a potential conflict of interest.

## Publisher’s note

All claims expressed in this article are solely those of the authors and do not necessarily represent those of their affiliated organizations, or those of the publisher, the editors and the reviewers. Any product that may be evaluated in this article, or claim that may be made by its manufacturer, is not guaranteed or endorsed by the publisher.
